# Estrogen associates with female predominance in Xp11.2 translocation renal cell carcinoma

**DOI:** 10.1038/s41598-023-33363-0

**Published:** 2023-04-15

**Authors:** Yanwen Lu, Yiqi Zhu, Wenliang Ma, Ning Liu, Xiang Dong, Qiancheng Shi, Fei Yu, Hongqian Guo, Dongmei Li, Weidong Gan

**Affiliations:** 1 Department of Urology, Nanjing Drum Tower Hospital, The Affiliated Hospital of Nanjing University Medical School, No. 321 Zhongshan Road, Nanjing, 210008 Jiangsu People’s Republic of China; 2 Department of Laboratory Medicine, Nanjing Children’s Hospital, The Affiliated Hospital of Nanjing Medical University, Nanjing, Jiangsu People’s Republic of China; 3 Immunology and Reproduction Biology Laboratory and State Key Laboratory of Analytical Chemistry for Life Science, Medical School, Nanjing University, Nanjing, Jiangsu People’s Republic of China; 4 Jiangsu Key Laboratory of Molecular Medicine, Nanjing University, Nanjing, Jiangsu People’s Republic of China

**Keywords:** Cancer epidemiology, Oncogenes, Urological cancer

## Abstract

Based on the epidemiological characteristics of susceptibility and age selectivity for women in Xp11.2 translocation renal cell carcinoma (Xp11.2 tRCC), we inferred that estrogen was to be blamed. Rad54 like 2 (*Rad54l2*) which might be one of key effector proteins of DNA damage mediated by estrogen was downregulated in numerous cancers, however, its role in epidemiological characteristics of Xp11.2 tRCC was needed to further study. We reviewed 1005 Xp11.2 tRCC cases and collected estrogen data and then compared the onset time of Xp11.2 tRCC cases in female with estrogen changing trend. An RNA-sequencing was performed in estrogen treated HK-2 cells and subsequently bioinformatic analysis was applied based on the Cancer Genome Atlas (TCGA) and GEO database. The male-to-female ratio of Xp11.2 tRCC was 1:1.4 and the median age of onset was 29.7 years old. The onset trend of female was similar to estrogen physiological rhythm (r = 0.67, *p* < 0.01). In Xp11.2 tRCC and HK-2 cells after estrogen treatment, Rad54l2 was downregulated, and GSEA showed that pathways significantly enriched in DNA damage repair and cancer related clusters after estrogen treated, as well as GO and KEGG analysis. Downregulation of Rad54l2 was in numerous cancers, including renal cell carcinoma (RCC), in which Rad54l2 expression was significantly decreased in male, age over 60 years old, T2&T3&T4 stages, pathologic SII&SIII&SIV stages as well as histologic G3&G4 grades, and cox regression analysis proved that Rad54l2 expression was a risk factor for overall survival, disease-specific survival and progression-free interval in univariate analysis. There existed female predominance in Xp11.2 tRCC and Rad54l2 might play vital role in estrogen mediating female predominance in Xp11.2 tRCC.

## Introduction

Xp11.2 translocation renal cell carcinoma (Xp11.2 tRCC) was firstly defined as a distinctive renal cell carcinoma (RCC) entity in 2004^1^ and then reclassified into microphthalmia (MiT) family tRCC in 2016^2^. This category of RCC is caused by breakage of the transcription factor enhancer 3 (*TFE3*) gene on X-chromosome and then balanced translocation with partner genes, such as *ASPL, PRCC, SFPQ, NonO* and *CLTC*^3–10^.

Xp11.2 tRCC was firstly reported in a two-year-old child^11^. Recent literatures showed that Xp11.2 tRCC occurred mostly in young people^12^ and the median age of onset is 29 years old^13^. However, in non-Xp11.2 tRCC, the median age is in the sixth and seventh decades^14^. A meta-analysis had revealed that the incidence of female is higher than that of male^15^, which is similar to the previous report by Cheng et al*.*^16^. In contrast, large-scale epidemiological investigation show that RCC is more common in male regardless of pathological type with a male-to-female ratio of 2:1^17^. Therefore, female predominance in childbearing period may be a noteworthy feature of Xp11.2 tRCC according to the age and gender data.

The gender disparity in RCC had been shown to be associated with sex steroids^18,19^. Preceding literature had also reported that estrogen could increase risk of topoisomerase IIβ-mediated *TFE3* breaks to initiate Xp11.2 tRCC^20^. This epidemiological feature may suggest that estrogen is a risk factor of Xp11.2 tRCC.

Moreover, androgen and androgen receptor could facilitate Xp11.2 tRCC progression^21^. Estrogen influenced the DNA damage response (DDR) and DNA repair through the regulation of key effector proteins, such as ATR, ATRX, Rad51, Rad54b^22^. Rad54 like 2 (*Rad54l2*), a paralog of DNA repair and recombination protein *Rad54b*, participated in super-helical torsion within linear DNA fragments in an ATP-dependent manner^23^. It was widely involved in replication and DNA repair^24^, previous study also reported that Rad54l2 was downregulated after radiotherapy, indicating impaired DNA repair capacity^25^. However, no systemic investigation into its role was carried out in the induction of Xp11.2 tRCC.

In this study, firstly, we demonstrated female predominance in Xp11.2 tRCC, and the onset trend of female was similar to estrogen physiological rhythm. Then, we explored the expression of Rad54l2 in Xp11.2 tRCC and HK-2 cells after estrogen treatment with RNA-sequencing and bioinformatic analysis to study the role of estrogen in Xp11.2 tRCC. Finally, we investigated Rad54l2 expression in many malignant tumors, including RCC and evaluated its role in RCC through bioinformatic analysis. These findings may offer a novel perspective to explain that estrogen was to be blamed for the female predominance of Xp11.2 tRCC.

We present the following article in accordance with the MDAR reporting checklist.

## Materials and methods

### Literature search and study selection

We performed a nonsystematic review in Cochrane, DARE, MEDLINE, EMBASE and PubMed using key words “Xp11.2 translocation”, “*TFE3*” and “renal cell carcinoma”. Since Xp11.2 tRCC was first reported in 1986, articles published from 1 January 1986 to 31 December 2020 was selected. All listed articles were reviewed carefully, in case of some cases repeated and left out. The diagnosis of Xp11.2 tRCC was based on: (1) strong TFE3 nuclear positivity by immunohistochemical staining; (2) Positive results in TFE3 break-apart FISH; (3) Confirmation by RNA-sequencing or karyotyping.

### Estrogen data collection

We collected estrogen data from Affiliated Nanjing Drum Tower Hospital of Medical School of Nanjing University and Nanjing Children’s Hospital. The inclusion criteria were as followed: (1) healthy people; (2) Estrogen level was not influenced. Finally, estrogen data from 1800 patients were enrolled in this study. The serum concentration of estrogen from the 1800 samples were tested by chemiluminescent immunoassay. The study protocol was approved by the ethics committee of Affiliated Nanjing Drum Tower Hospital of Medical School of Nanjing University and Nanjing Children’s Hospital and conformed to the principles of the Declaration of Helsinki. Data collection was approved by the institutional review board and performed in accordance with the ethical standards established by Nanjing Drum Tower Hospital of Medical School of Nanjing University and Nanjing Children’s Hospital. Written informed consent was obtained from all included samples before the clinical investigations were performed.

### RNA-sequencing

HK-2 cells treated with 10 nmol/L estrogen for 24 h were harvested, then total RNA was isolated by using TRIzol reagent (Vazyme, R401) following the manufacturer's procedure. The RNA amount and purity of each sample was quantified with RIN number > 7.0, OD260/280 > 1.8, total RNA > 1 μg. At last, we performed the 2 × 150 bp paired-end sequencing (PE150) on an illumina Novaseq™ 6000 (LC-Bio Technology CO., Ltd., Hangzhou, China) following the vendor's recommended protocol.

### Bioinformatics analysis of RNA-seq

Fastp software (https://github.com/OpenGene/fastp) were used to make quality control for raw data. HISAT2 (https://ccb.jhu.edu/software/hisat2) were used to map reads to the reference genome of Homo sapiens GRCh38. We used StringTie (https://ccb.jhu.edu/software/stringtie) with default parameters to assemble the mapped reads. After the final transcriptome was generated, StringTie was used to estimate the expression levels of all transcripts and mRNAs by calculating FPKM. We denied the differentially expressed mRNAs with fold change > 2 or fold change < 0.5 and with parametric F-test comparing nested linear models (p value < 0.05) by R package edgeR (https://bioconductor.org/packages/release/bioc/html/edgeR.html). Gene set enrichment analysis (GSEA) was operated according to the given gene set from the Molecular Signatures Database (MSigDB) (http://software.broadinstitute.org/gsea/msigdb). Gene Ontology (GO) and Kyoto Encyclopedia of Genes and Genomes (KEGG) were analyzed by R package “clusterProfiler”.

### Cell culture and reagent

The cell line HK-2, 786-O was purchased from ATCC. UOK109 and UOK120 were a gift of Marston Linhan from NIH. All cells were maintained in 90% Dulbecco's Modified Eagle Medium (Gibco, 10569010) supplemented with 10% fetal bovine serum (Gibco, 16140063) and 1% penicillin/streptomycin (Invitrogen, 15070063) in 5% CO_2_ at 37 °C. E2 (Sigma-Aldrich, E8875) was dissolved in DMSO (Sigma-Aldrich, D2650).

### RNA isolation and quantitative real-time PCR

TRIzol extraction reagent (Vazyme, R401) was used to isolate total RNA which was then reverse-transcribed using a Reverse Transcriptase master mixing kit (Vazyme, R201) according to the manufacturer's procedure. Primers (18s rRNA forward primer: CAGCCACCCGAGATTGAGCA, 18s rRNA reverse primer: TAGTAGCGACGGGCGGTGTG; Rad54l2 forward primer: AGGAGTGTGACAGGGATGATG; Rad54l2 reverse primer: TCCTCGGAGGCTAGGTTCTTG; ER-α forward primer: AGATCTTCGACATGCTGCTGGCTA, ER-α reverse primer: AGACTTCAGGGTGCTGGACAGAAA; ER-β forward primer: TGGGCACCTTTCTCCTTTAGTGGT, ER-β reverse primer: TCTTGCTTCACACCAGGGACTCTT) were synthesiszed by Tsingke Biological Technology. PCR amplifications were operated by using SYBR Green (Vazyme, Q711) and ABI ViiA7 System (Applied Biosystems). Relative expression of target gene was calculated by the comparative2 ^–(ΔΔCt)^ method normalizing on 18S rRNA.

### Data mining the GEO database

Rad54l2 mRNA expression and clinical data were from GEO database (GSE167573) and (GSE150474), in GSE167573, there were 63 Xp11.2 tRCC cases and 14 normal cases. 12 Xp11.2 tRCC cases and 7 normal cases were included in GSE150474. Ethical approval was not required, as all are open to the public. High-throughput sequencing and clinical data were analyzed using R software (version 3.6.3).

### Statistical analysis

According to the distribution of demographic characteristics, all cases were grouped by a 5-year interval. Then we calculated the number of cases and average estrogen levels in each group. Taking age as abscissa, the number of male and female cases and the variation trend of estrogen with age were plotted respectively. Unpaired t-test was used to analyze the significance of cases among different age groups by GraphPad Prism software version 8.0 (GraphPad Software, San Diego, CA). The correlation analysis was used to evaluate the relationship between female cases and physiological rhythm of estrogen levels by Origin 2018 (OriginLab, Northampton, Massachusetts, USA). Student's t-test was used to assess differences in Rad54l2 mRNA expression between different groups. Kaplan–Meier curve was performed to evaluate the survival status using the log-rank test. Receiver operator characteristic (ROC) curve were drawn to assess the prognostic value of Rad54l2. Univariate and multivariate analyses were performed using a Cox proportional hazard regression model A two-tailed p-value of < 0.05 was considered to be statistically significant.

## Results

### Similar trends between the onset trend of female and estrogen physiological rhythm in Xp11.2 tRCC

**Figure 1:**
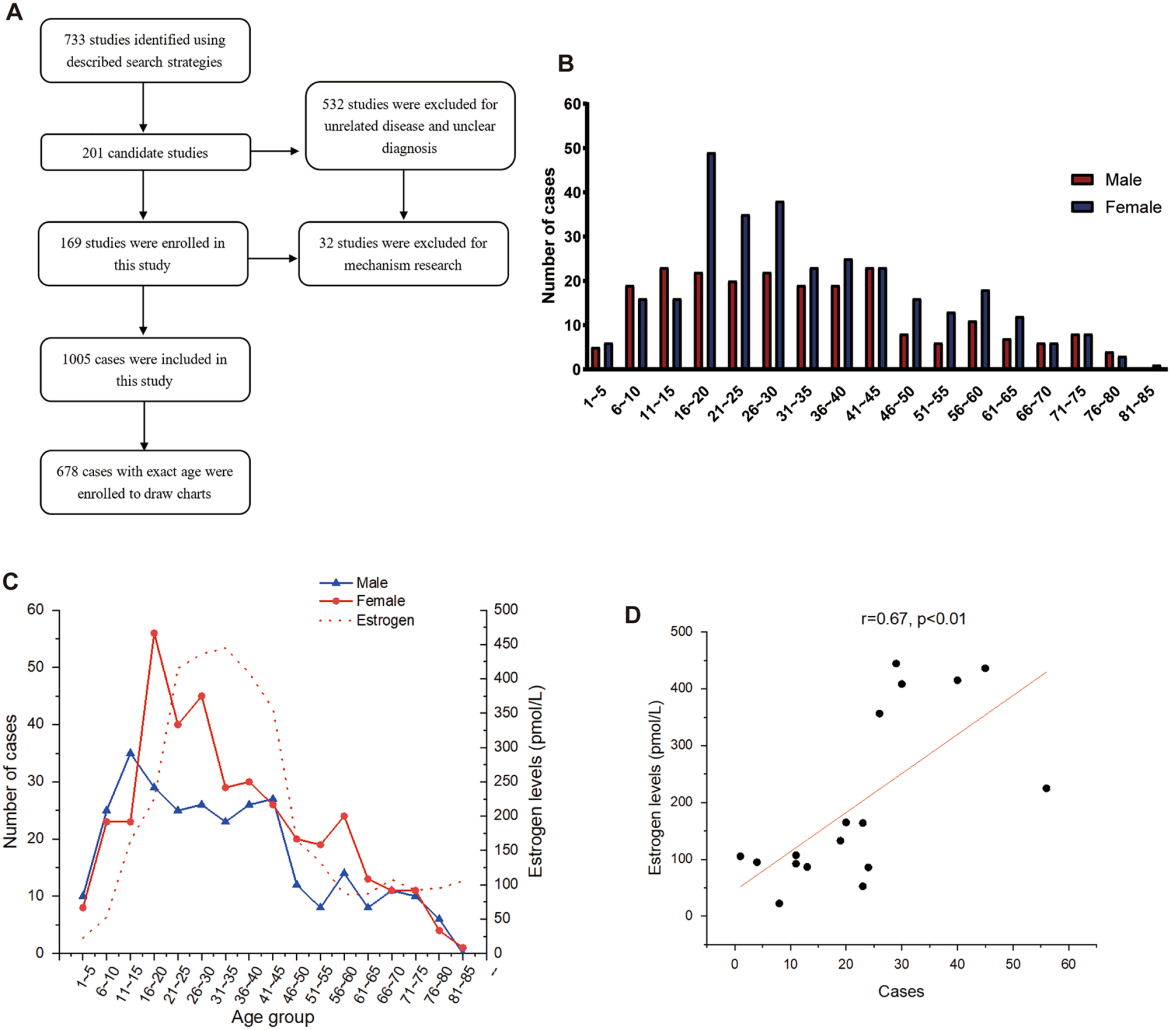
Similar trends between the onset trend of female and estrogen physiological rhythm in Xp11.2 tRCC. (**A**) Flow diagram of study selection. (**B**) The gender distribution in different age groups. (**C**) The trend of the onset time of Xp11.2 tRCC cases in female and estrogen levels. (**D**) Correlation analysis of the trends of female cases and estrogen changing trend with age. n.s.: p ≥ 0.05; *p < 0.05; **p < 0.01; ***p < 0.001; ****p < 0.0001.

After excluding the unqualified literature, a total of 169 articles fulfilled the inclusion criteria. Of 1005 enrolled Xp11.2 tRCC patients, 417 were men and 584 were women (male-to-female ratio = 1:1.4). 295 male cases and 383 female cases with exact age of onset from initially 1005 cases were selected, with male-to-female ratio = 1:1.3. Overall, the age of onset ranged from 1 to 85 years old with median age approximately 29.7 years old (Fig. 1A,B). There were 124 cases under 16 years old, accounting for 18.29%. 382 cases were at the age of 16–45 during child-bearing period, which made up of 56.53%. After 45 years old, there were 172 cases with the proportion of 25.37% (Table 1). For trend analysis, the difference in sex ratio can be divided into three stages and the results were shown in Table 1. From childhood to adolescence, the number of Xp11.2 tRCC cases was consistently raised. Before the age of 16, the incidence of Xp11.2 tRCC in women was similar to that in men (p > 0.05) with the male-to-female ratio of 1.3:1. Between 16 to 45 years old, the number of male cases remained stable, however, the quantity of female cases increased sharply, with a peak value in age group of 16–20 years old. The number of female cases was more than that of male significantly (p < 0.05), with male-to-female ratio = 1:1.45. After 45 years old, especially after 66 years old, the quantity of male cases was approximately equal to female cases (p > 0.05) (Fig. 1C). Estrogen levels rose from childhood and reached to a peak value in age group of 21–25 years old, then remained at a high level until the age of 45. This result was consistent with the physiological changes of estrogen levels in female. In addition, there was a significant correlation (r = 0.67, p < 0.01) between the number of female cases and the changes of estrogen (Fig. 1D).

**Table 1:** Gender disparity between male and female in different age groups.

Age group	Males (n = 295)	Females (n = 383)	p value
0–15	10	8	> 0.05
	25	23	
	35	23	
16–45	29	56	< 0.05
	25	40	
	26	45	
	23	29	
	26	30	
	27	26	
46–85	12	20	> 0.05
	8	19	
	14	24	
	8	13	
	11	11	
	10	11	
	6	4	
	0	1	

### Rad54l2 was downregulated in HK-2 cell treated with estrogen and Xp11.2 tRCC

**Figure 2:**
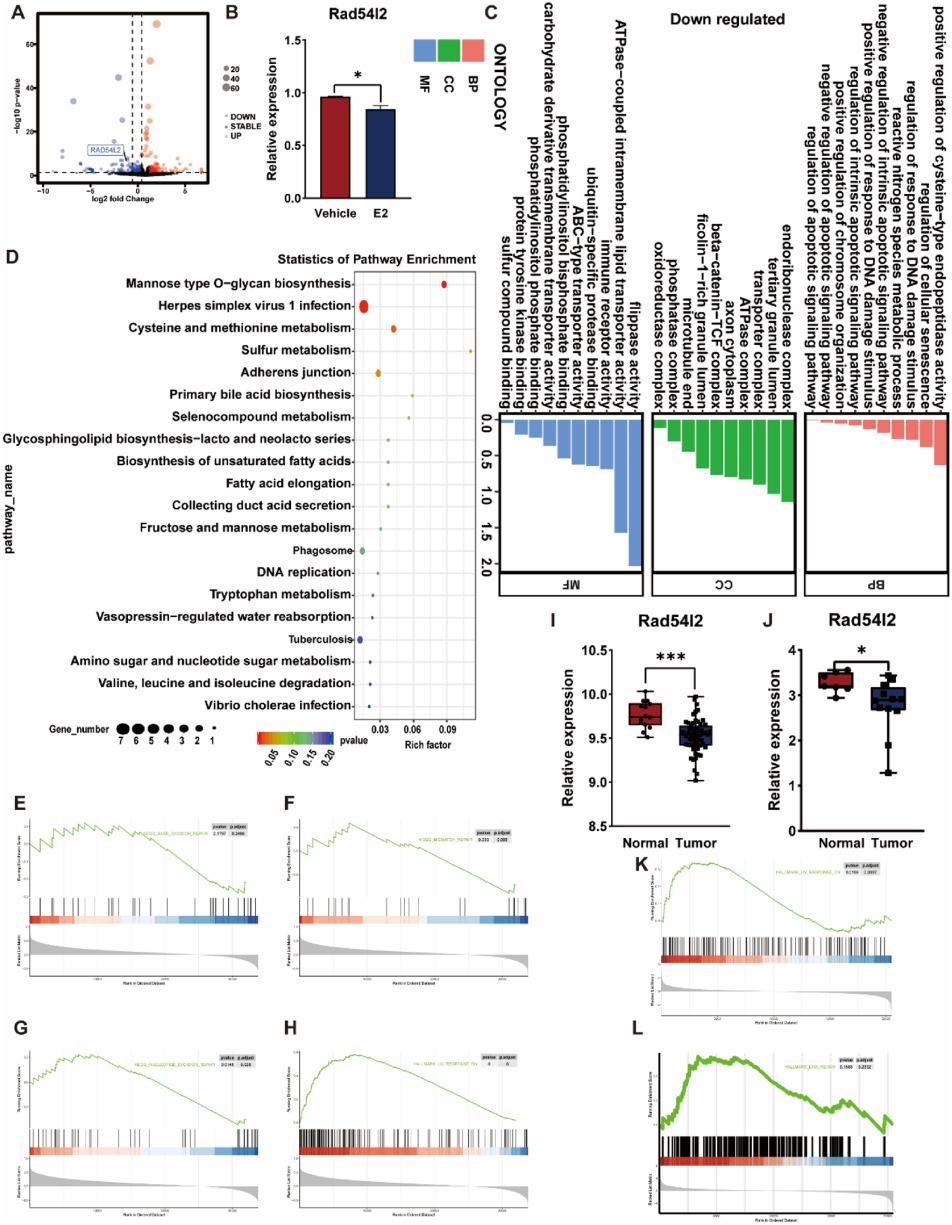
Rad54l2 was downregulated in HK-2 treated with estrogen and Xp11.2 tRCC. (**A**) RNA-sequencing found that Rad54l2 was downregulated after estrogen treatment. (**B**) qRT-PCR further confirmed this result. (**C**,**D**) GO and KEGG analysis enriched in DNA damage repair related clusters in RNA-sequencing. (**E**–**H**) GSEA enrichment analysis in DNA damage repair related clusters in RNA-sequencing. (**I**,**J**) Rad54l2 mRNA was downregulated in Xp11.2 tRCC. Data from (GSE167573) and (GSE150474) respectively. (**K**,**L**). GSEA analysis enriched in UV response down in Xp11.2 tRCC. Data from (GSE167573).

**Figure 3:**
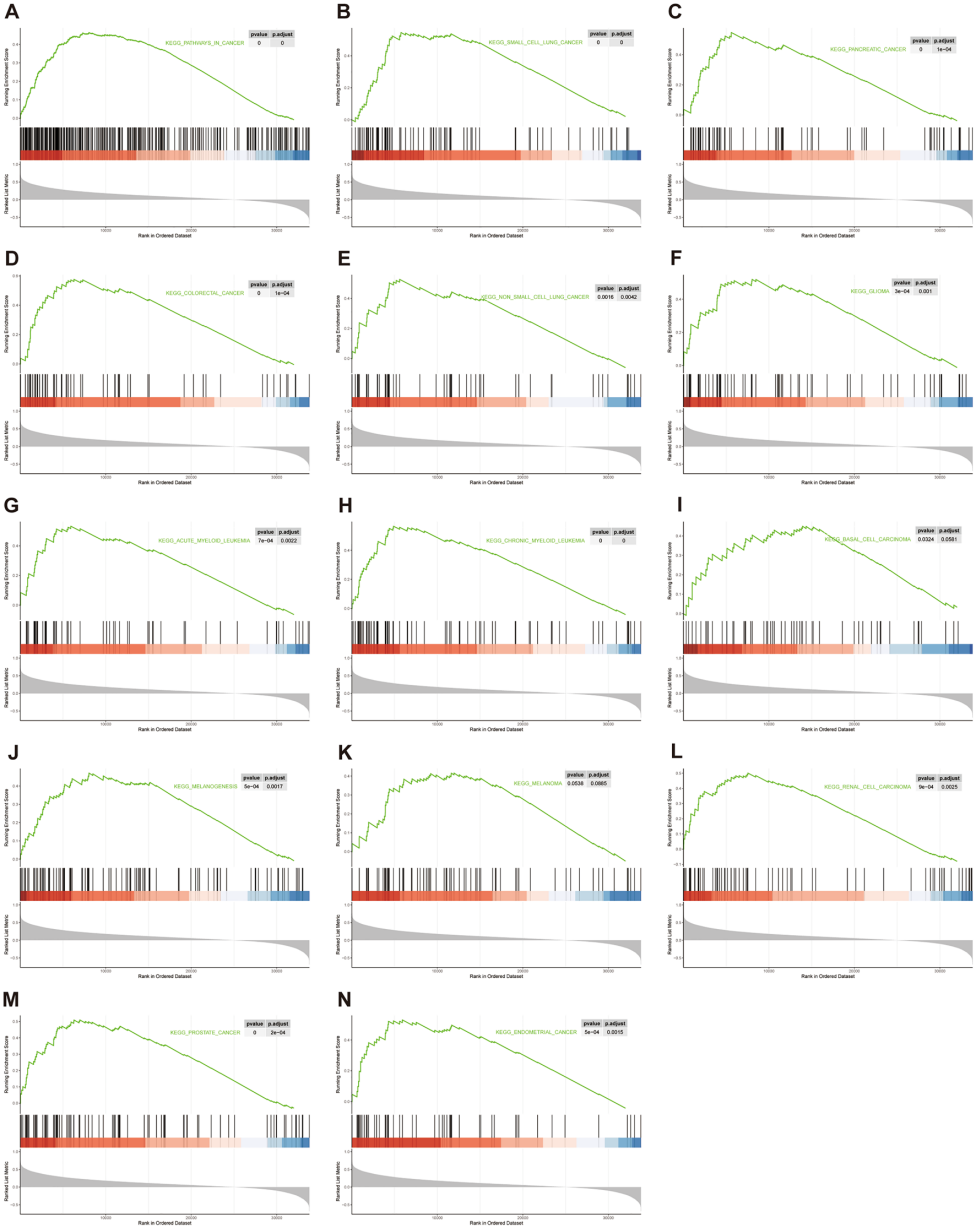
Cancer related clusters were enriched in GSEA analysis. Pathways in (**A**) cancer, (**B**) small cell lung cancer, (**C**) pancreatic cancer, (**D**) colorectal cancer, (**E**) non-small cell lung cancer, (**F**) glioma, (**G**) acute myeloid leukemia, (**H**) chromic myeloid leukemia, (**I**) basal cell carcinoma, (**J**) melanogenesis, (**K**) melanoma as well as for (**L**) renal cell carcinoma, in addition, (**M**) endometrial cancer and (**N**) prostate cancer were significantly enriched as well.

Previous study in our center demonstrated that estrogen induced *TFE3* breakage dependent on ER in the renal tubular epithelial cell HK-2 cell^20^, however, which molecule mediated this process was uncovered. Therefore, an RNA-sequencing which after HK-2 cell was treated with estrogen 24 h at a concentration of 10 nM was conducted. It was verified that Rad54l2 was significantly downregulation after estrogen treatment among DNA repair related genes (Fig. 2A), whereafter qRT-PCR further confirmed this result (Fig. 2B). It was suggested that some pathways were enriched, including positive regulation of response to DNA damage stimulus and regulation of response to DNA damage stimulus in GO and DNA replication in KEGG analysis (Fig. 2C,D). In addition, according to GSEA, Rad54l2-associated differentially expressed genes were enriched in DNA damage repair related clusters, involved in base excision repair, mismatch repair, nucleotide excision repair and UV response down (Fig. 2E–H). Results from GEO database (GSE167573) and (GSE150474) observed that Rad54l2 was also dramatically downregulation when compared with normal tissue in Xp11.2 tRCC (Fig. 2I,J), as well as, GSEA enriched significantly in UV response down cluster and DNA repair pathway (Fig. 2K,L). GAEA analysis found that after HK-2 cell treatment with estrogen, Rad54l2-associated differentially expressed genes were also enriched in cancer pathway, including small cell lung cancer, pancreatic cancer, colorectal cancer, non-small cell lung cancer, glioma, acute myeloid leukemia, chromic myeloid leukemia, basal cell carcinoma, melanogenesis, melanoma (Fig. 3A–K), as well as for RCC (Fig. 3L), in addition, endometrial cancer and prostate cancer were also significantly enriched (Fig. 3M,N), which partial caused by hormone in oncogenesis.

### Expression profiles of Rad54l2 in numerous cancers

**Figure 4:**
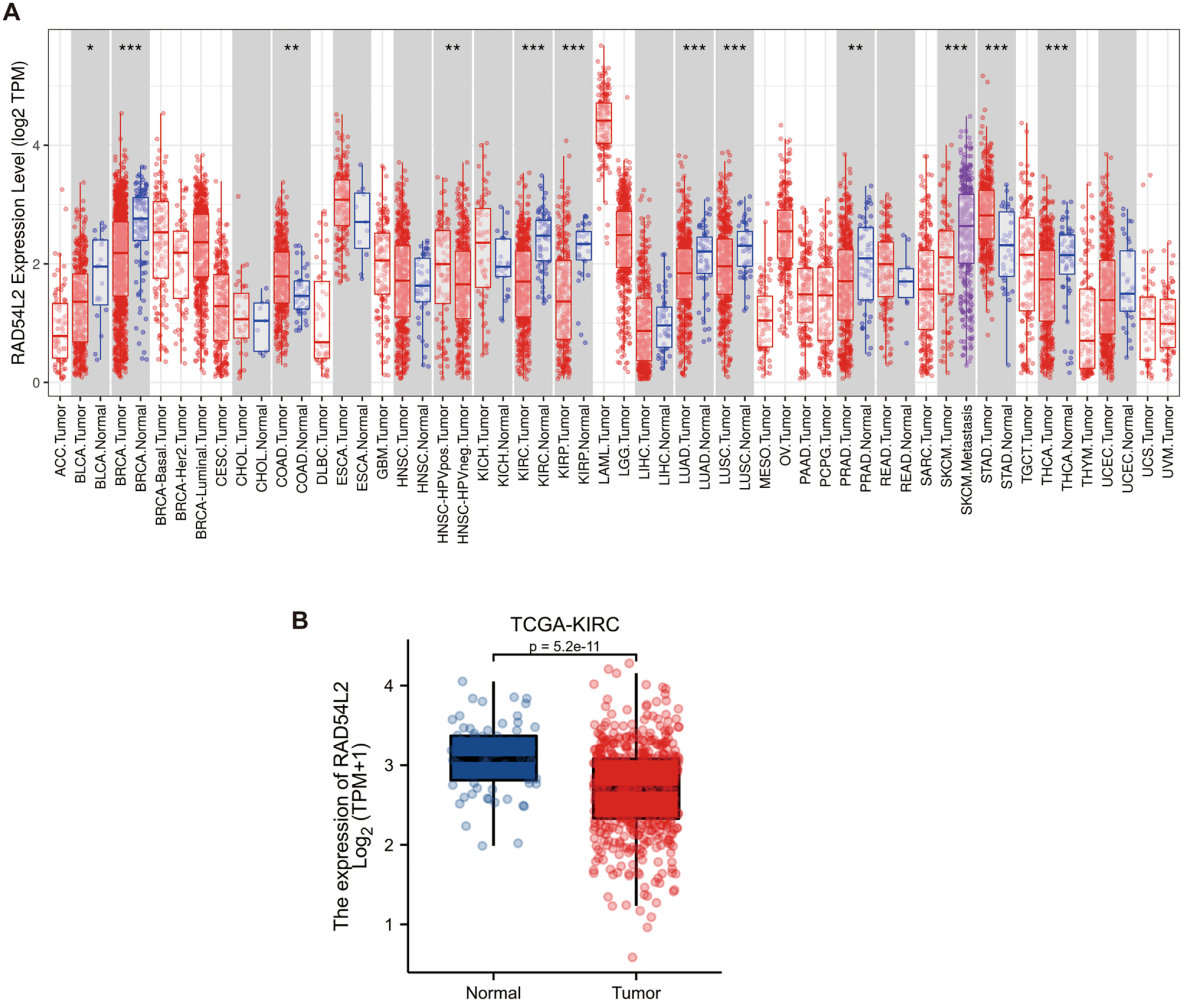
Expression profiles of Rad54l2 mRNA in numerous cancers. (**A**) Rad54l2 expression in a plenty of cancers between tumor and tumor-adjacent tissue. (**B**) Rad54l2 is higher than pericarcinous tissue in RCC.

We utilized the cancer genome atlas (TCGA) online database to determine the mRNA of Rad54l2. As shown in (Fig. 4A), among 38 cancer types, the Rad54l2 was significantly low-expressed in 10 cancers, except for stomach adenocarcinoma. Moreover, Rad54l2 expression was much lower in KRIC tumors than in normal tissues (P < 0.001, Fig. 4B). Interestingly, expression of Rad54l2 in skin cutaneous melanoma metastasis is higher than that in primary sites.

### Association of Rad54l2 expression and clinicopathological characteristics in RCC patients

**Figure 5:**
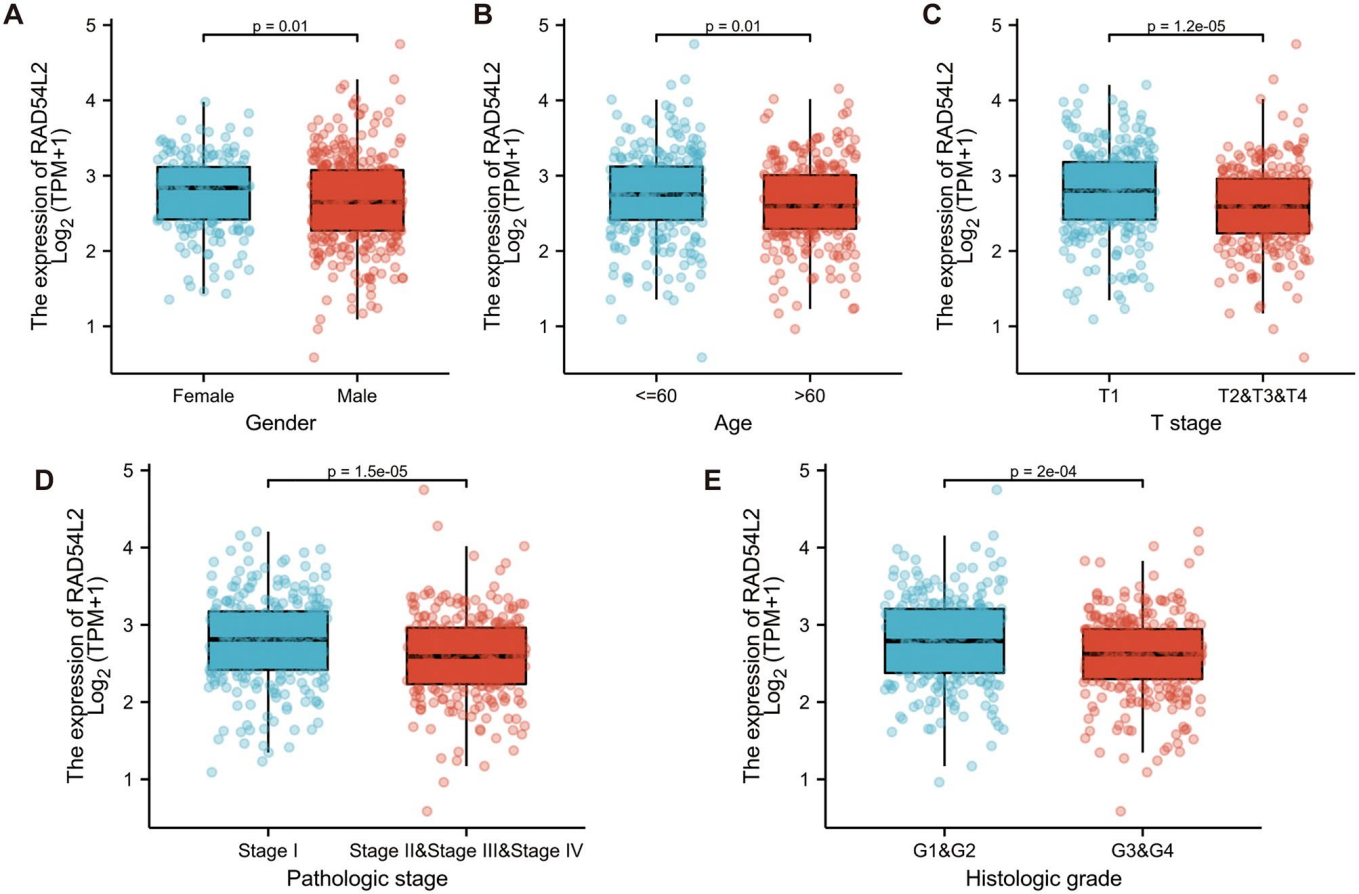
Association of Rad54l2 Expression and Clinicopathological Characteristics in RCC Patients. The association of Rad54l2 Expression with (**A**) Gender, (**B**) Age, (**C**) T stage, (**D**) Pathologic stage, (**E**) Histologic grade was analyzed.

Clinicopathological characteristics of RCC with differentially expressed Rad54l2 were collected, as shown in Table 2. Patients in Rad54l2-low group presented a higher percent of male sex, older age, much more severe clinical T stage, worse pathologic stage and poorer histologic grade when compared to the Rad54l2-high group. Nevertheless, clinical N stage and M stage were without significant difference between two groups. Furthermore, we analyzed Rad54l2 expression in patients with different clinicopathological characteristics. As shown in (Fig. 5A–E), Rad54l2 expression was significantly decreased in male sex, age over 60 years old, T stages T2, T3 and T4, pathologic stages Stage II, Stage III and Stage IV, histologic grade Grade3 and Grade 4.

**Table 2:** Clinicopathological characteristics of RCC patients with differential Rad54l2 expression.

Characteristic	Low expression of RAD54L2	High expression of RAD54L2	p
n	265	265	
Gender, n (%)			0.005
Female	77 (14.5%)	109 (20.6%)	
Male	188 (35.5%)	156 (29.4%)	
Age, n (%)			0.019
≤ 60	118 (22.3%)	146 (27.5%)	
> 60	147 (27.7%)	119 (22.5%)	
T stage, n (%)			0.020
T1	118 (22.3%)	153 (28.9%)	
T2	39 (7.4%)	30 (5.7%)	
T3	103 (19.4%)	76 (14.3%)	
T4	5 (0.9%)	6 (1.1%)	
N stage, n (%)			1.000
N0	115 (45.1%)	124 (48.6%)	
N1	8 (3.1%)	8 (3.1%)	
M stage, n (%)			0.711
M0	208 (41.8%)	212 (42.6%)	
M1	41 (8.2%)	37 (7.4%)	
Pathologic stage, n (%)			0.011
Stage I	115 (21.8%)	150 (28.5%)	
Stage II	30 (5.7%)	27 (5.1%)	
Stage III	75 (14.2%)	48 (9.1%)	
Stage IV	44 (8.3%)	38 (7.2%)	
Histologic grade, n (%)			0.004
G1	2 (0.4%)	12 (2.3%)	
G2	103 (19.7%)	124 (23.8%)	
G3	113 (21.6%)	93 (17.8%)	
G4	44 (8.4%)	31 (5.9%)	

*RCC* renal cell carcinoma.

### Predictive value of Rad54l2 for RCC diagnosis and prognosis

**Figure 6:**
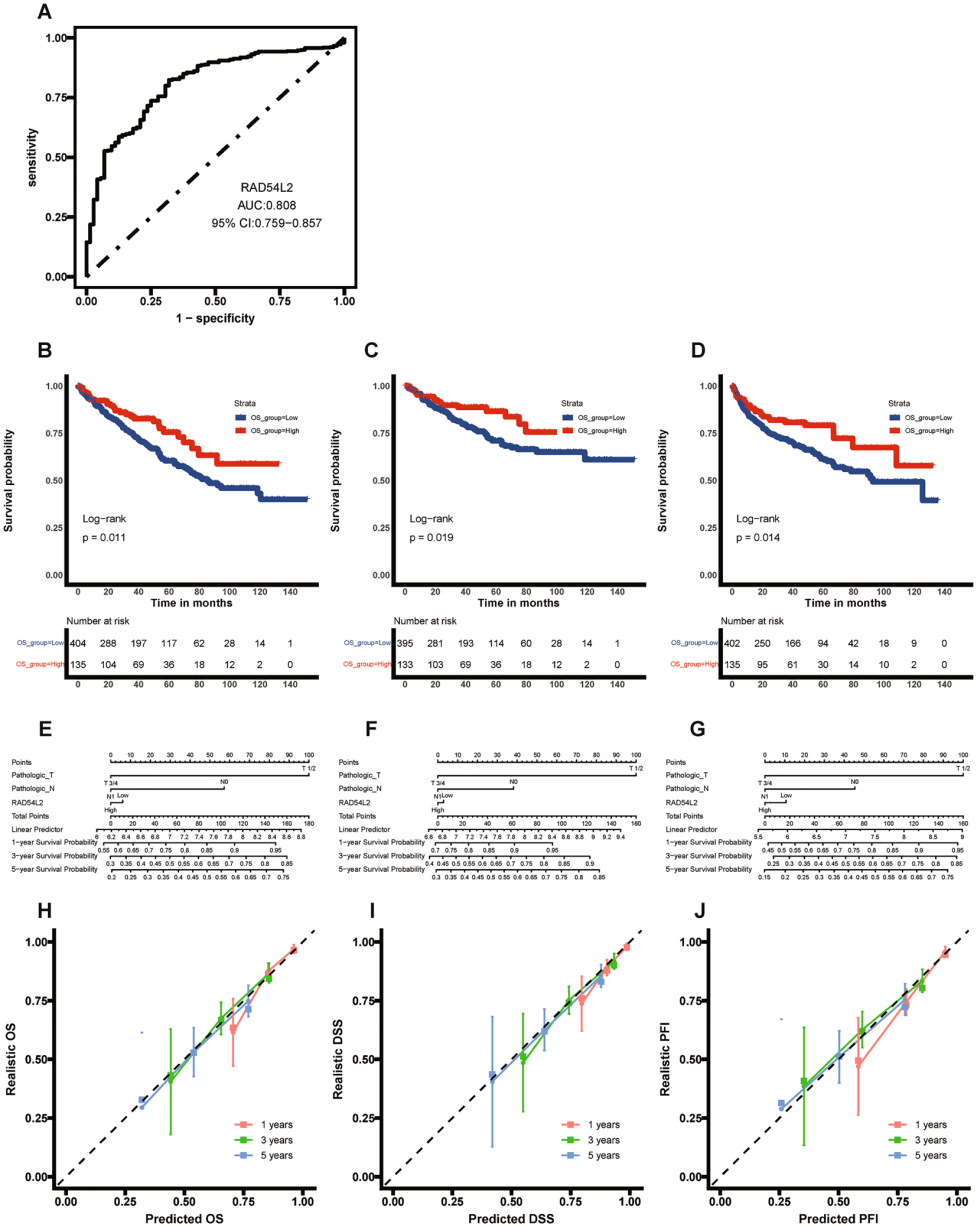
Predictive value of Rad54l2 for RCC diagnosis and prognosis. (**A**) The value of Rad54l2 for RCC diagnosis was analyzed by Receiver operating characteristic (ROC) curve. (**B**–**D**) K–M curve for OS, DSS and PFI between Rad54l2-high and Rad54l2-low groups. (**E**–**G**) Rad54l2-based risk scoring models for 1-, 3-, and 5-year OS, DSS and PFI were constructed in nomograms. (**H**–**J**) Calibration curves were drawn to test the efficiency of the corresponding nomograms for OS, DSS and PFI.

ROC curve analysis was used to analyze the power of Rad54l2 in the differential diagnosis of RCC patients. Based on the area under curve (AUC) being 0.808, Rad54l2 showed significant high sensitivity and specificity for RCC diagnosis (Fig. 6A). Subsequently, we utilized K-M analysis to demonstrate the role of Rad54l2 in RCC prognosis. It is suggested that overall survival (OS), disease-specific survival (DSS) and progression-free interval (PFI) for low-Rad54l2 group were all significantly poorer than high-Rad54l2 group (Fig. 6B–D). As well as, we conducted a cox regression analysis to further confirm the forecast value of Rad54l2 on prognosis. As shown in Table 3, Rad54l2 expression was a risk factor for OS, DSS and PFI. A prognostic nomogram was drawn by using all the statistically significant prognostic factors in each cox regression analysis. The nomogram consisted of T stages, M stages and Rad54l2 expression (Fig. 6E–G). Then, we drawn a calibration curve was to verify the efficiency of each nomogram. All the curves shown dramatically favorable prediction of the nomogram for the 1-, 3-, and 5-year clinical outcomes (Fig. 6H–J).

**Table 3:** Cox regression analysis for clinical outcomes in RCC patients.

Characteristics	HR for overall survival	HR for progression-free interval	HE for disease-specific survival
Age (> 60 vs. ≤ 60)	1.80****	1.33	1.40
Sex (male vs. female)	0.93	1.52*	1.22
Stage (stage III/IV vs. stage I/II)	3.95****	6.82****	9.83****
Histological grade (grade III/IV vs. stage I/II)	2.70****	3.65****	4.79****
T stage (T3–T4 vs. T1–T2)	3.23****	4.52****	5.54****
N stage (N1–N2 vs. N0)	3.45****	3.68****	3.85****
M stage (M1 vs. M0)	4.39****	8.97****	9.11****
Smoker (yes vs. no)	0.79	1.06	1.22
RAD54L2 (high vs. low)	0.60*	0.60*	0.54*

*HR* hazard ratio; *p < 0.05; ****p < 0.0001.

## Discussion

In the past few decades, sex hormones have been shown to have a certain effect on the development and progression of RCC^26,27^. However, the effect of sex hormone in Xp11.2 tRCCs was not evaluated. The present study which conducted with more than 1000 Xp11.2 tRCC cases was the largest case collection to date. The results showed that the overall male-to-female ratio of Xp11.2 tRCC was 1:1.4, which is different from the male-to-female ratio of incidence in conventional RCC. Interestingly, this female advantage mainly occurred during 16–45 years old, when estrogen was maintained at a high level in women. The starting and ending time of female advantage of incidence in Xp11.2 tRCC corresponded to the estrogen level in adolescence and menopause period, respectively, indicating estrogen physiological rhythm was correlated to female predominance during child-bearing period.

We observed that Rad54l2 was downregulation in HK-2 cells after estrogen treatment and Xp11.2 tRCC. In addition, GSEA showed that pathways significantly enriched in DNA damage repair and cancer related clusters after estrogen treated. Go and KEGG analysis also got similar results. Rad54l2 expression in RCC, was significantly decreased in male, age over 60 years old, T2&T3&T4 stages, pathologic SII&SIII&SIV stages as well as histologic G3&G4 grades, and cox regression analysis proved that Rad54l2 expression was a risk factor for OS, DSS and PFI in univariate analysis.

Defects in DNA repair pathway are key steps for tumorigenesis, incorrectly repaired DNA breaks could result in chromosome instability and progression to cancer^28^. Rad54l2 binds to DNA and mononucleosomes and involves in chromatin remodeling, DNA excision repair and homologous recombination^29^, whose reduction would result in impaired DNA repair, thus chromosome translocation. Previous study has demonstrated that irradiation also downregulates Rad54l2, accompanied by γH2A.X increment, which indicates DNA damage occurred^25^.

It is suggested that estrogen is a complete carcinogen, being capable of tumor formation by suppressing DNA repair to allow the accumulation of genomic change conducive to tumorigenesis^22^. Estrogen receptors (ERs) are discovered in both normal kidney tissue and kidney cancer^30^, indicating estrogen works in kidney cancer. It is proved that ERα could regulate negatively ATM and ATR which are key initiators of DDR^31^. In addition, ERα could also impact on DNA repair through interacting directly with various DNA repair proteins, including FEN1, MPG, APE1, and TDG of the BER pathway, NHEJ repair proteins Ku70 as well as Ku86, MSH2 of the MMR pathway^32^.

Xp11.2 tRCC is characterized by *TFE3* gene breakage on X chromosome. For tumor involving X chromosome, the number advantage of X chromosome is a probable cause of female predominance^33^. However, synovial sarcoma with balanced translocation t(X;18), which pathogenesis is similar to Xp11.2 tRCC, showed male predominance^34^. In addition, alveolar soft part sarcoma which involves with unbalanced translocation of *TFE3* showed similar incidence rate between male and female^35^. These results indicated that female predominance did not always appear in cancers involving the X chromosome. Therefore, the number of X chromosome could not completely explain the sex difference in morbidity.

It is reported that prior exposure to TOP2 poison may induce DNA double-strand breaks (DSBs) and thus result in Xp11.2 tRCC formation^36,37^. Many animal models also demonstrated that estrogen can induce microsatellite DNA alterations and DNA single-strand breaks in Syrian hamsters and led to kidney cancer^26,38–41^, which is similar to the impact of estrogen in breast cancer^42,43^. Accumulated evidences support that estrogen metabolites, 2-hydroxy-estrogen (2-OHE2) and 16α-hydroxyoestrone (16α-OHE1) generate reactive oxygen species^44–46^, resulting in DNA breakage. Among a number of diseases related to estrogen metabolism, many show female predominance similarity with Xp11.2 tRCC. For instance, pulmonary arterial hypertension (PAH) has a similar incidence trend with Xp11.2 tRCC, with male-to-female ratio 1:1.7, mean age 36.4 years old^47^. It is suggested that the ratio of 2-OHE2/16α-OHE1 can influence PAH attack rate^47–49^. In addition, the increase of Estrogen-DNA adduct makes female more susceptible to Fuchs endothelial corneal dystrophy and lung cancer^50,51^.

Our results should be interpreted cautiously. For the rarity of Xp11.2 tRCC, case reports were also included and our search items were likely to be inadequate for all cases included, in addition, clinicopathological data form RCC were applied to confirm the role of Rad54l2 in diagnostic and prognosis value, which might seem to be unreasonable. Considering the low incidence of Xp11.2 tRCC and the lack of effective preoperative diagnostic methods as well as estrogen data from Xp11.2 tRCC were scarcely possible to obtain, so we conducted the analyses using estrogen data from patients with estrogen levels uninfluenced. We were inclined to believe that estrogen through Rad54l2 caused this female predominance of Xp11.2 tRCC, but this hypothesis should be proven by further studies.

In conclusion, this population-based study gives a new insight to the sex differences in Xp11.2 tRCC. The change trend of estrogen during different periods is in accordance with the curve of the onset age in female. Rad54l2 was also shown diagnostic and prognosis value for RCC. The downregulated Rad54l2 in Xp11.2 tRCC and HK-2 with estrogen treatment might be a key factor for estrogen mediating chromosome translocation. These findings suggest that estrogen is a potential pathogenic factor of Xp11.2 tRCC although more molecular evidences are needed.

## Supplementary Information

Supplementary Information.

## Data Availability

The RNA-sequencing data analyzed in this study are not publicly available but can be obtained from the corresponding author.
