# Supplementary material for: Estrogen associates with female predominance in Xp11.2 translocation renal cell carcinoma
Source: Sci Rep. 2023 Apr 15;13:6141. doi: 10.1038/s41598-023-33363-0 (PMC10105720; doi:10.1038/s41598-023-33363-0)
Supplement: Supplementary file 1 — Supplementary Information. [file 41598_2023_33363_MOESM1_ESM.docx]

Cox regression analysis for overall survival in RCC patients.

| Characteristics | Total(N) |  | Multivariate analysis | |
| --- | --- | --- | --- | --- |
|  |  |  | Hazard ratio (95% CI) | P value |
| RAD54L2 | 541 |  |  |  |
| High | 271 |  |  |  |
| Low | 270 |  | 1.045 (0.678 - 1.610) | 0.841 |
| Pathologic stage | 538 |  |  |  |
| Stage III&Stage IV | 206 |  |  |  |
| Stage I&Stage II | 332 |  | 0.800 (0.314 - 2.038) | 0.639 |
| Histologic grade | 533 |  |  |  |
| G3&G4 | 283 |  |  |  |
| G1&G2 | 250 |  | 0.580 (0.356 - 0.944) | **0.028** |
| Pathologic T stage | 541 |  |  |  |
| T3&T4 | 191 |  |  |  |
| T1&T2 | 350 |  | 0.644 (0.284 - 1.461) | 0.292 |
| Pathologic N stage | 258 |  |  |  |
| N1 | 16 |  |  |  |
| N0 | 242 |  | 0.585 (0.293 - 1.169) | 0.129 |
| Pathologic M stage | 508 |  |  |  |
| M1 | 79 |  |  |  |
| M0 | 429 |  | 0.388 (0.226 - 0.668) | **< 0.001** |

Cox regression analysis for disease-specific survival in RCC patients.

| Characteristics | Total(N) |  | Multivariate analysis | |
| --- | --- | --- | --- | --- |
|  |  |  | Hazard ratio (95% CI) | P value |
| RAD54L2 | 530 |  |  |  |
| High | 264 |  |  |  |
| Low | 266 |  | 1.285 (0.744 - 2.218) | 0.368 |
| Pathologic stage | 527 |  |  |  |
| Stage III&Stage IV | 198 |  |  |  |
| Stage I&Stage II | 329 |  | 0.358 (0.118 - 1.090) | 0.071 |
| Histologic grade | 522 |  |  |  |
| G3&G4 | 273 |  |  |  |
| G1&G2 | 249 |  | 0.496 (0.254 - 0.968) | **0.040** |
| Pathologic T stage | 530 |  |  |  |
| T3&T4 | 183 |  |  |  |
| T1&T2 | 347 |  | 0.745 (0.321 - 1.732) | 0.495 |
| Pathologic N stage | 256 |  |  |  |
| N1 | 15 |  |  |  |
| N0 | 241 |  | 0.679 (0.311 - 1.482) | 0.331 |
| Pathologic M stage | 497 |  |  |  |
| M1 | 75 |  |  |  |
| M0 | 422 |  | 0.262 (0.140 - 0.489) | **< 0.001** |

Cox regression analysis for progression-free interval in RCC patients.

| Characteristics | Total(N) |  | Multivariate analysis | |
| --- | --- | --- | --- | --- |
|  |  |  | Hazard ratio (95% CI) | P value |
| RAD54L2 | 539 |  |  |  |
| High | 270 |  |  |  |
| Low | 269 |  | 0.932 (0.590 - 1.470) | 0.761 |
| Pathologic stage | 536 |  |  |  |
| Stage III&Stage IV | 204 |  |  |  |
| Stage I&Stage II | 332 |  | 0.270 (0.109 - 0.669) | **0.005** |
| Histologic grade | 531 |  |  |  |
| G3&G4 | 281 |  |  |  |
| G1&G2 | 250 |  | 0.563 (0.338 - 0.937) | **0.027** |
| Pathologic T stage | 539 |  |  |  |
| T3&T4 | 189 |  |  |  |
| T1&T2 | 350 |  | 1.046 (0.511 - 2.138) | 0.903 |
| Pathologic N stage | 257 |  |  |  |
| N1 | 16 |  |  |  |
| N0 | 241 |  | 0.754 (0.377 - 1.507) | 0.424 |
| Pathologic M stage | 506 |  |  |  |
| M1 | 77 |  |  |  |
| M0 | 429 |  | 0.249 (0.145 - 0.429) | **< 0.001** |


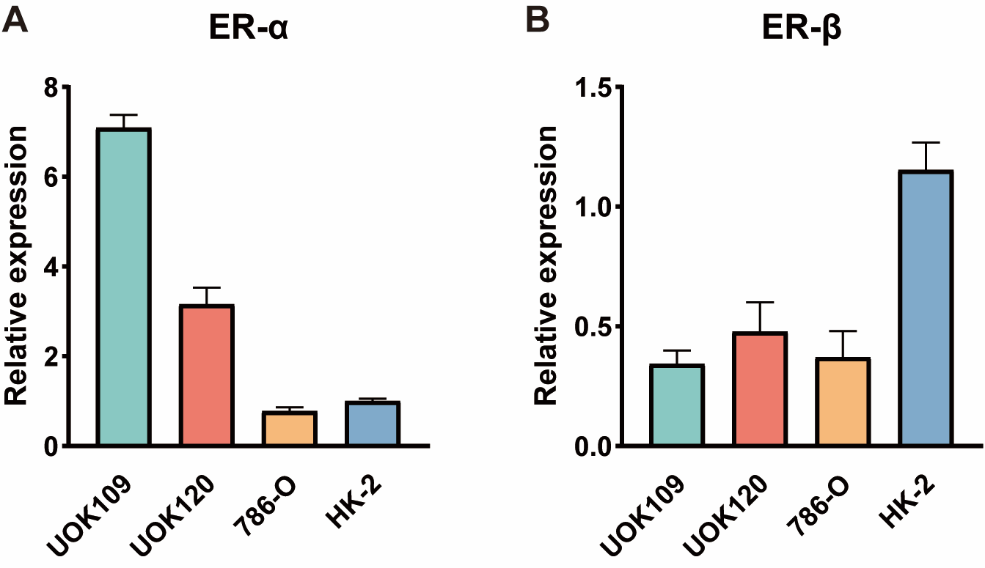


**Supplementary Figure.** ER expression in HK-2 cell. (A-B). qRT-PCR was performed to detect the expression of ER-α and ER-β in UOK109, UOK120, 786-O and HK-2.
